# Colonic TRPV4 overexpression is related to constipation severity

**DOI:** 10.1186/s12876-023-02647-0

**Published:** 2023-01-13

**Authors:** Hiroshi Mihara, Kunitoshi Uchida, Yoshiyuki Watanabe, Sohachi Nanjo, Miho Sakumura, Iori Motoo, Takayuki Ando, Masami Minemura, Jibran Sualeh Muhammad, Hiroyuki Yamamoto, Fumio Itoh, Ichiro Yasuda

**Affiliations:** 1 Center for Medical Education and Career Development, Graduate School of Medicine and Pharmaceutical Sciences, University of Toyama, Toyama, Japan; 2 Department of Gastroenterology, Graduate School of Medicine and Pharmaceutical Sciences, University of Toyama, Toyama, Japan; 3 Department of Physiological Science and Molecular Biology, Fukuoka Dental College, Fukuoka, Japan; 4 Department of Internal Medicine, Kawasaki Rinko General Hospital, Kawasaki, Japan; 5 Department of Basic Medical Sciences, College of Medicine, University of Sharjah, Sharjah, United Arab Emirates; 6 Division of Gastroenterology and Hepatology, Department of Internal Medicine, St. Marianna University School of Medicine, Kawasaki, Japan; 7 Department of Bioinformatics, St. Marianna University Graduate School of Medicine, Kawasaki, Japan

**Keywords:** Colon, TRPV4, *E. faecalis*, *E. coli*, Chronic constipation

## Abstract

**Background:**

Chronic constipation is prevalent and involves both colon sensitivity and various changes in intestinal bacteria, particularly mucosa-associated microflora. Here we examined regulatory mechanisms of TRPV4 expression by co-culturing colon epithelial cell lines with intestinal bacteria and their derivatives. We also investigated TRPV4 expression in colon epithelium from patients with constipation.

**Methods:**

Colon epithelial cell lines were co-cultured with various enterobacteria (bacterial components and supernatant), folate, LPS, or short chain fatty acids. TRPV4 expression levels and promoter DNA methylation were assessed using pyrosequencing, and microarray network analysis. For human samples, correlation coefficients were calculated and multiple regression analyses were used to examine the association between clinical background, rectal TRPV4 expression level and mucosa-associated microbiota.

**Results:**

Co-culture of CCD841 cells with *P. acnes*, *C. perfringens*, or *S. aureus* transiently decreased TRPV4 expression but did not induce methylation. Co-culture with clinical isolates and standard strains of *K. oxytoca*, *E. faecalis*, or *E. coli* increased TRPV4 expression in CCD841 cells, and TRPV4 and TNF-alpha expression were increased by *E. coli* culture supernatants but not bacterial components. Although folate, LPS, IL-6, TNF-alpha, or SCFAs alone did not alter TRPV4 expression, TRPV4 expression following exposure to *E. coli* culture supernatants was inhibited by butyrate or TNF-alphaR1 inhibitor and increased by p38 inhibitor. Microarray network analysis showed activation of TNF-alpha, cytokines, and NOD signaling. TRPV4 expression was higher in constipated patients from the terminal ileum to the colorectum, and multiple regression analyses showed that low stool frequency, frequency of defecation aids, and duration were associated with TRPV4 expression. Meanwhile, incomplete defecation, time required to defecate, and number of defecation failures per 24 h were associated with increased *E. faecalis* frequency.

**Conclusions:**

Colon epithelium cells had increased TRPV4 expression upon co-culture with *K. oxytoca*, *E. faecalis*, or *E. coli* supernatants, as well as TNFα-stimulated TNFαR1 expression via a pathway other than p38. Butyrate treatment suppressed this increase. Epithelial TRPV4 expression was increased in constipated patients, suggesting that TRPV4 together with increased frequency of *E. faecalis* may be involved in the pathogenesis of various constipation symptoms.

**Supplementary Information:**

The online version contains supplementary material available at 10.1186/s12876-023-02647-0.

## Background

Chronic constipation is a high-frequency disease that has a 5% prevalence, especially in the elderly population. Stimulation of the rectum induces defecation [1], but 23% of patients with constipation, especially those with defecation disorder, have low rectal sensitivity [2, 3], suggesting that dysfunction of intestinal sensitivity is involved in chronic constipation pathogenesis. Functional diseases of the lower gastrointestinal tract are related to gut microbiota. Previous studies showed that prevalence of Actinobacteria (e.g., *Bifidobacterium*, *acnes*) and Firmicutes (e.g., *Lactobacillus*, *Staphylococcaceae*, *Clostridium*) is decreased in the elderly, whereas Protozoa, Bacteroidetes and Proteobacteria (e.g., *E. coli*) are increased [4]. In terms of intestinal bacterial changes in constipation, the transit time of the distal colon is correlated with intestinal microbiota diversity [5]. Prolonged intestinal transit time in young and middle-aged women in Europe and the United States is associated with a decrease in Prebotella spp. [6]. There are gender differences in intestinal bacteria in the Japanese population, and this diversity was not related to the stool shape scale. *Oscillospira spp*. is increased in hard stools, and *Bifidobacterium* is significantly more abundant in stools from women who experience constipation [7, 8]. In the mucosa-associated flora of constipated patients, Bacteroidetes increased (Flavobacteriaceae increased, Odoribacteraceae decreased) and *Bifidobacterium* and *Faecalibacterium* decreased [9].

TRPV4 (transient receptor potential channel vanilloid 4) is a nonselective cation channel that is activated by mechanical stimuli, hypo-osmotic pressure, heat, and epoxyeicosatrienoic acid. TRPV4 is also sensitized by PAR-2, 5-HT, and histamine [10, 11]. Dental pulp cells stimulated by TNFα exhibit TRPV4 enhancement via the TNFR1 and p38 MAPK signaling pathways [12], whereas in articular cartilage TRPV4 enhancement occurs via the Erk and p38 MAPK signaling pathways [13, 14]. However, the mechanisms that regulate TRPV4 activity in the gastrointestinal epithelium are unknown. Butyrate is known to inhibit induction of TNFα expression [15, 16]. We previously showed that TRPV4 is present throughout the gastrointestinal epithelium, including the colon [17], and is associated with physiological or abnormal gastrointestinal motility and perception. We also found that in the gastric epithelium of patients with *H. pylori* TRPV4 expression was silenced by DNA methylation and the expression was restored upon *H. pylori* eradication [18]. In colon epithelial cells, folate and LPS were shown to be involved in methylation of various genes [19–22]. In the present study, we aimed to elucidate the intracellular signals involved in TRPV4 expression in colonic epithelium. Like *H. pylori* in the stomach, methylation of TRPV4 is suppressed by intestinal bacteria and increased by treatment with TNFα. Here we investigated how changes in TRPV4 expression in colonic epithelium from healthy subjects and patients with chronic constipation were related to constipation symptoms and populations of mucosal-adhesive intestinal bacteria.

## Methods

### Cell line and culture conditions

CCD 841 CoTr cells (ATCC Cat# CRL-1807, RRID:CVCL_2872) were cultured in accordance with the manufacturer’s instructions. The cells were maintained in a humidified incubator at 33 °C. We previously reported that CCD841 cells express functionally active TRPV4 [23].

### Co-culture conditions

In this study, we evaluated 12 representative clinical isolates of enterobacteria, followed by 10 standard isolates of enterobacteria that are known to alter TRPV4 expression (Additional file 2: Table S1). The list of intestinal bacteria associated with constipation is not exhaustive, but for this study we selected intestinal bacteria that have been suggested to be associated with constipation whenever possible. The clinical isolates were obtained from the Department of Clinical Laboratory, Toyama University Hospital, and the standard strains were obtained from the RIKEN Microbiological Materials Development Laboratory or from the Research Institute for Microbial Diseases, Osaka University. Aerobic bacteria were cultured in Bacto Tryptone (1–7 days), and anaerobic bacteria were cultured in GAM Semisolid "Nissui" under microaerobic conditions (5% O_2_, 10% CO_2_, 85% N_2_, 37 °C; Sanyo-Multigas Incubator; Sanyo Electric Co. Tokyo, Japan), 100% humidity, and 160 rpm in a gyratory shaker (Thermo Shaker; Thermonix, Tokyo, Japan). The equation of absorbance 0.1 = 10^8^ cells/mL was used to estimate the concentration of bacteria in each culture medium. Co-culture of CCD 841 cells with bacterial strains was performed as described previously with some modifications [24]. Briefly, CCD 841 cells were seeded into 6 cm culture dishes and incubated for 24 h as described above before the cultures were washed three times with phosphate-buffered saline (PBS). Fresh medium without antibiotics or FBS was added one hour before adding the bacteria. Bacteria were first cultured under the above conditions and then washed twice with PBS before direct addition to CCD 841 cell cultures at a bacteria/cell ratio of 50:1. The co-cultures were incubated for the indicated times. For preparation of bacterial supernatants and bacterial components, bacterial suspensions having an O.D. value of 0.1 (10^8) were added to the cell culture medium and incubated for 1 day before the supernatant and the bacteria were collected. The bacterial components were made by heating bacterial suspensions at 100 °C for 20 min. After heating, the bacteria were cultured again to confirm that inactivation was complete. The culture supernatant was filtered through a 0.22um filter and diluted 50% with fresh culture medium since undiluted supernatants were toxic to cells.

### Chemicals

Unless otherwise noted, the following compounds were from Sigma (St. Louis, MO) and the concentrations and treatment times used are listed: IL-6 (Funakoshi, Japan, 2.5 ng/ml for 6 h or 24 h) [25], TNF-alpha (1–100 ng/ml for 1 h to 24 h), folate (100 μM), LPS (1 pg/ml–20 g/ml) [26], butyrate, acetate, propionate (100 μM) [27]; TNF-a receptor 1 (TNFR1) inhibitor R 7050 (5 μmol/mL) and p38 inhibitor SB202190 (10 or 100 μM).

### Reverse transcription PCR analysis

Experiments were conducted as previously reported [18]. Total RNA (1 µg) was isolated using an RNeasy Mini Kit (Qiagen, Hilden, Germany). Quantitative RT (qRT)-PCR was performed using the QuantiFast SYBR Green PCR Kit (QIAGEN) with the 7300 Real-time PCR System (Applied Biosystems, CA, USA) or iQ5 (Bio-Rad Laboratories, CA, USA). Cycling conditions were 94 °C for 5 min followed by 40 cycles of 94 °C for 15 s and 60 °C for 30 s. Data were collected and analyzed as values relative to β-actin. Primer information is provided in Additional file 2: Table S2.

### Western blotting

Western blotting was performed as previously described [28] using the antibodies summarized in Additional file 2: Table S3. CCD841 cell lysates were resolved by SDS-PAGE on 7.5% SDS–polyacrylamide gels and transferred to polyvinylidene membranes. The membranes were cut, blocked with BlockAce and probed with primary antibodies. Immunopositive bands were visualized with the ECL system (Thermo Fisher Scientific, MA, USA) and an ImageQuant LAS4000 (GE Healthcare) instrument. TRPV4 and β-Actin band densities were measured using ImageJ software (https://imagej.nih.gov/ij/).

### Quantitative DNA methylation analysis

DNA was extracted using an AllPrep DNA/RNA Mini Kit (QIAGEN, Valencia, CA). Bisulfite conversion of DNA was performed using an EpiTect bisulfite kit (QIAGEN, Valencia, CA). Biotinate Polymerase Chain Reaction was performed using primers that specifically amplify TRPV4 gene sequences (Additional file 2: Table S4). PCR assays included a denaturation step at 95 °C for 30 s, followed by an annealing step at various temperatures for 30 s, and an extension step at 72 °C for 30 s. Pyrosequencing was performed using PSQ HS 96 Gold single-nucleotide polymorphism reagents with a Pyromark Q24 pyrosequencing machine (Biotage, Uppsala, Sweden). The protocol for pyrosequencing has been previously described in detail [29]. Pyrosequencing quantitatively measures the methylation status of several CpG sites in a given promoter. These adjacent sites usually show highly concordant methylation. Therefore, the mean percentage of methylation of the detected sites was used as a representative value for each gene promoter.

### Gene expression microarrays and data analysis and filter criteria

cRNA was amplified, labeled, and hybridized to a 60 K Agilent 60-mer oligomicroarray according to the manufacturer's instructions. An Agilent scanner was used to scan all hybridized microarray slides. Relative hybridization intensities and background hybridization values were calculated using Agilent Feature Extraction Software (9.5.1.1). Raw signal intensities and Flags for each probe were calculated from hybridization intensities (gProcessedSignal), and spot information (e.g., gIsSaturated), according to the procedures recommended by Agilent. (Flag criteria on GeneSpring Software: Absent (A): “Feature is not positive and significant” and “Feature is not above background”; Marginal (M): “Feature is not Uniform”, “Feature is Saturated”, and “Feature is a population outlier”; Present (P): others). Furthermore, the raw signal intensities of two samples were log_2_-transformed and normalized using a quantile algorithm with the ‘preprocessCore’ library package [30] in Bioconductor software [31]. We selected probes that had a ‘P’ flag in at least one sample and excluded lincRNA probes. To identify genes with up or down-regulated expression, we calculated Z-scores [32] and ratios (non-log scaled fold-change) based on the normalized signal intensities for each probe for comparison between control and experimental samples. Then, we established criteria for regulated genes with Z-score ≥ 2.0 and ratio ≥ 1.5-fold indicating up-regulated expression and Z-score ≤ − 2.0 and ratio ≤ 0.66 indicating down-regulated expression. Analysis of the microarray network with the KEGG database was performed using DAVID (https://david.ncifcrf.gov/home.jsp) [33]. The datasets generated and analyzed for the current study are available in the GEO repository (https://www.ncbi.nlm.nih.gov/geo/query/acc.cgi?acc=GSE208164).

### Patient background, rectal biopsy samples and mucosal adherent bacteria

Patients with constipation (ROME IV criteria) [34] and patients who underwent medical checkups at the University of Toyama Hospital were included in the study. Patient background (age, gender), Bristol stool shape scale (BS) and constipation severity scores (CSS) were recorded. Mucosal biopsy (terminal ileum, cecum, sigmoid colon and rectum) specimens and mucosa-associated intestinal bacteria were collected with a brush (Orcellex, Medical & Biological Laboratories, Japan) at the time of colonoscopy and stored in RNAlater (QIAGEN, German). The CSS consisted of the following 8 items: (1) frequency of bowel movements, (2) painful evacuation, (3) incomplete evacuation, (4) abdominal pain, (5) length of time per attempt, (6) assistance for evacuation, (7) unsuccessful evacuation attempts over 24 h, and (8) duration of constipation [35] and slow transit constipation and/or defecation disorder; for most of the cases these items were complications.

### Mucosa-associated Enterobacteriaceae ratio

Bacteria-specific primers are listed in Additional file 2: Table S5 [36–42]. The ratios of *P. acnes*, *E. coli*, *K. oxytoca*, *C. perfringens*, *E. faecalis*, and *S. aureus* to 16 srRNA were calculated, and the correlation coefficients between the factors were calculated, followed by a multiple regression analysis. In addition, correlation with the ratios of both O and H antigens of *E. coli* was examined by antigen-specific real-time PCR using multimix primers [43].

### Data analysis and statistics

The qRT-PCR data are presented as the mean ± SEM of three or more independent experiments. Student's t-test was used to determine significance, with *p* < 0.05. As the data for the two groups were not equally distributed, Welch's method was used for the t-test. In examination of clinical specimens, the sample size was calculated as 10 times the number of independent variables to be entered. A total of 40 patients were included, with 3 healthy subjects for every 1 patient. Correlation coefficients for each item were calculated using the nonparametric Spearman method, and items related to CSS subscores (CSS sub) were examined by multiple regression analysis (forced entry method) using SPSS Statistics 24.0 (IBM Corp.).

## Results

### CCD841 cells co-culture studies

**Fig. 1:**
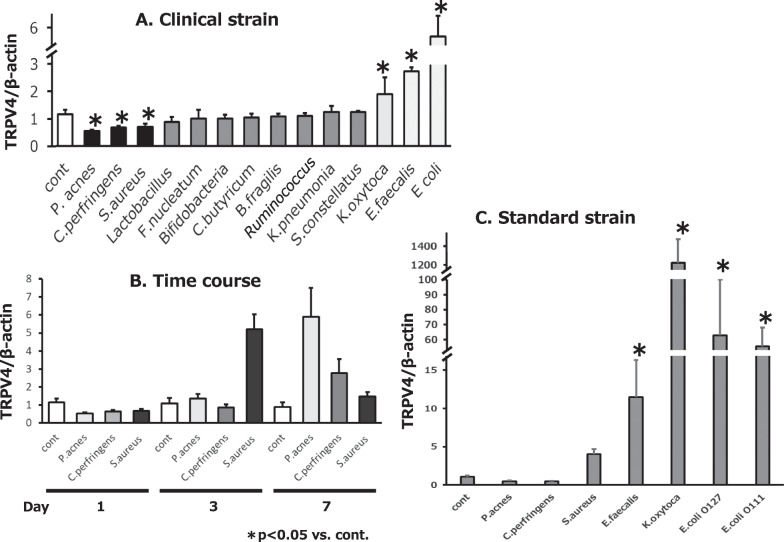
Effect of co-culturing CCD841 cells with bacterial suspensions on TRPV4 expression. **A** Decreased TRPV4 mRNA expression following 1 day co-culture of CCD841 cells with *P. acnes*, *C. perfringens* or *S. aureus* (clinical strain; black bars). Co-culture with *K. oxytoca*, *E. faecalis* or *E. coli* (white bars) for 7 days increased TRPV4 mRNA expression. Co-culture with the other types of bacteria (gray bars) did not affect TRPV4 expression. Asterisks indicate significant change in expression relative to untreated control cells. Error bars represent the mean ± S.E. of five to six trials. **B** For bacteria that decreased TRPV4 expression after one day of co-culture, suppression of TRPV4 expression returned after 3 days and 7 days of co-culture. **C** Increased TRPV4 expression in CCD841 cells co-cultured with *K. oxytoca*, *E. faecalis* or *E. coli* (standard strain) for 7 days

TRPV4 mRNA expression was significantly decreased in CCD841 intestinal epithelial cells co-cultured with clinical isolates of *P. acnes*, *C. perfringens*, or *S. aureus* for 1 day. Meanwhile, significant increases in TRPV4 expression were observed for co-culture with *K. oxytoca*, *E. faecalis*, and *E. coli* for 7 days. No change in expression was seen for co-culture with *Lactobacillus*, *F. nucleatum, Bifidobacteria*, *C. butyricum*, *B. fragilis* or *Ruminococcus* for 7 days (Fig. 1A). For those bacteria associated with increased expression, the increases occurred after 3 days of co-culture. For those with decreased expression, co-culture with *P. acne* or *C. perfringens* first decreased expression, which then began to increase after 3 days of co-culture and this increasing trend continued through 7 days, whereas for *S. aureus* co-culture the decrease began at 7 days (Fig. 1B). Next, the same experiment was performed with the standard strains of intestinal bacteria that were known to alter TRPV4 expression and a similar trend was observed (Fig. 1C).

### Pyrosequencing to detect *TRPV4* gene methylation after bacterial exposure

We examined whether abnormalities in *TRPV4* gene promoter methylation occurred in CCD841 cells co-cultured for 1 day with *P. acnes, S. aureus* or C*. perfringens* that exhibited suppression of TRPV4 expression, but few changes in methylation were detected (Additional file 1: Fig. S1). As positive controls, methylation levels of DLD1 and HCT116 [44], two colon cancer cell lines known to have high methylation levels, were shown to be 87% and 92%, respectively.

### Bacterial supernatant increases TRPV4 and TNF-alpha expression

**Fig. 2:**
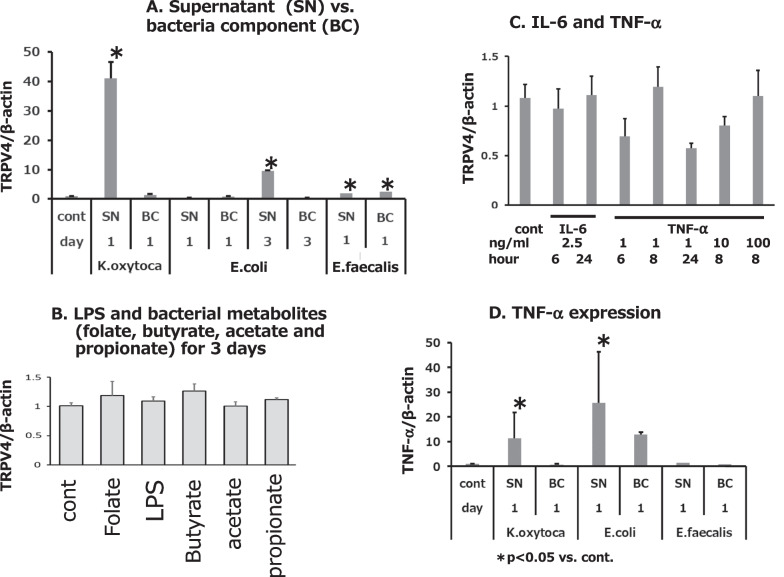
Effect of co-culture with bacterial supernatants or components on cytokine expression in CCD184 cells. **A** Exposure of CCD841 cells to *K. oxytoca* supernatant (SN) for 1 day increased TRPV4 expression, but co-culture with *K. oxytoca* bacterial components (BC) for 1 day did not. Exposure to *E. coli* (O111) SN for 3 days increased TRPV4 expression, but *E. coli* BC for 3 days did not. Exposure to either *E. faecalis* SN or BC for 1 day increased TRPV4 expression. **B** Treatment with folic acid, LPS (1 ng ~ 20 µg/ml), or SCFA (butyrate, acetate, and propionate) at any concentration for 3 days had no effect on TRPV4 expression in CCD841 cells. **C** Human IL-6 or TNF-alpha at any concentration or any duration (6, 8 or 24 h) did not affect TRPV4 expression in CCD841. **D** Exposure to *K. oxytoca* or *E. coli* SN for 1 day increased TNF-alpha expression in CCD841 cells, but *E. faecalis* SN and BC for 1 day did not. * indicates significant difference from untreated control. Error bars represent the mean ± S.E. of five to six trials

To determine whether the components that caused the increased TRPV4 expression were associated with bacterial supernatants or bacteria components themselves, we examined the effect of *K. oxytoca, E. coli* (O111) and *E. faecalis*. Co-culture of CCD841 cells with *K. oxytoca* (for 1 day co-culture) or *E. coli* (3 day co-culture) bacterial supernatants were both associated with significantly increased TRPV4 expression compared to treatment with the bacteria components. For *E. faecalis* (1 day), both bacterial components and bacterial supernatants slightly but significantly increased TRPV4 expression in CCD841 cells (Fig. 2A). Meanwhile, treatment with the bacterial metabolic products folic acid, LPS (1 ng ~ 20 µg/ml), or SCFA (butyrate, acetate, and propionate) for 3 days did not alter TRPV4 expression at any concentration (Fig. 2B). We also treated the cells with major cytokines (IL-6 and TNF-alpha) for 6 to 24 h and saw no change in TRPV4 expression (Fig. 2C). However, levels of TNF-alpha expression were significantly elevated when CCD841 cells were treated with culture supernatants of *K. oxytoca* (for 1 day) or *E. coli* (for 3 days), but not those of *E. faecalis* (Fig. 2D).

### Microarray network analysis in CCD841 cells responding to *E. coli* supernatant exposure

**Fig. 3:**
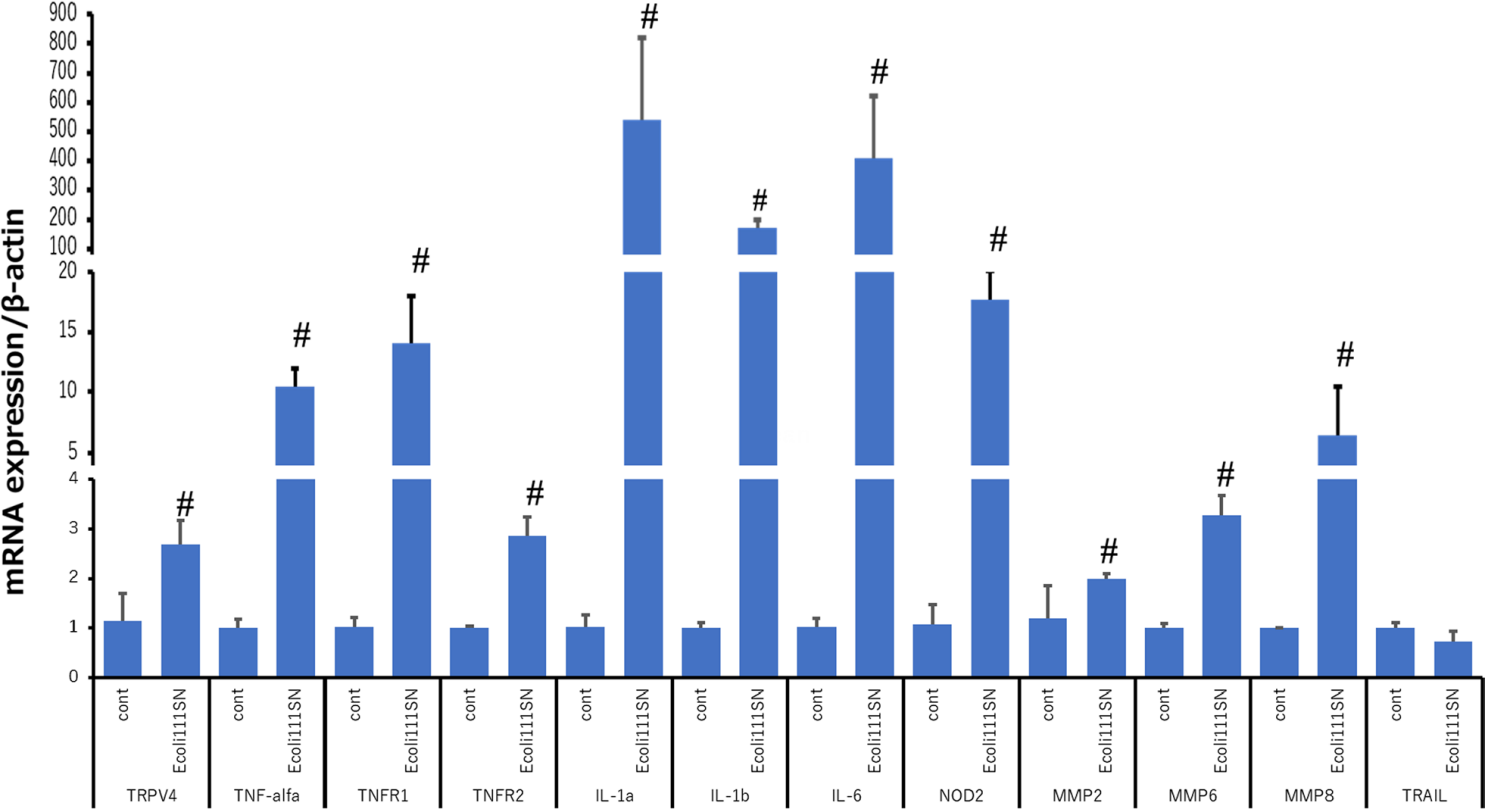
Bacterial supernatants affect gene expression in TNF-alpha signaling pathways. Exposure of CCD841 cells to *E. coli* (O111) supernatant (SN) increased relative amounts of TRPV4, TNF-alpha, TNFR1, TNFR2, IL-1, IL-6, NOD2, and MMP mRNA. # indicates significant difference (*p* < 0.05) between treated and untreated control cells. Error bars represent the mean ± S.E. of five to six trials

**Fig. 4:**
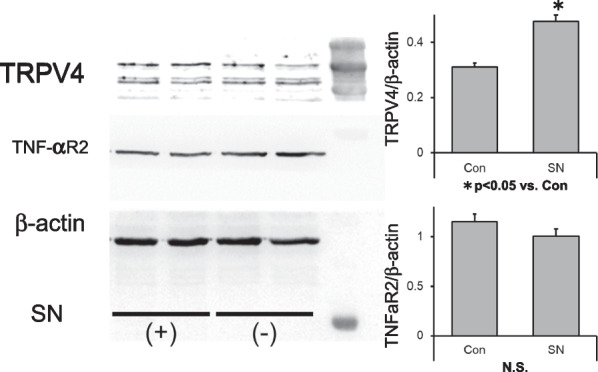
Effect of *E. coli.* O111 supernatant on TRPV4 protein expression in CCD841 cells. Western blot of CCD841 cells co-cultured with *E. coli* (O111) supernatant (SN) for 3 days shows a significant increase in TRPV4 protein expression relative to untreated control cells (*p < 0.05). Error bars represent the mean ± S.E. of five to six trials. The rightmost lane of the blot shows the ladder

CCD841 cells were treated with low concentrations (1 ml supernatant/3 ml medium) of *E. coli* (O-111) supernatant and a microarray network analysis using the KEGG pathway database was carried out. This analysis showed activation of TNF-alpha signaling, cytokines and NOD. Those pathways that exhibited significant changes are shown in Additional file 1: Fig. S2 and the list of highly expressed genes is presented in Additional file 2: Table S6. qRT-PCR follow-up of genes exhibiting increased expression confirmed the microarray results, although TNFαR1 levels were increased and only a small increase in TNFαR2 expression was observed (Fig. 3). Western blotting also showed significantly increased expression of TRPV4 at the protein level, with no clear increase in TNFαR2 protein (Fig. 4).

**Fig. 5:**
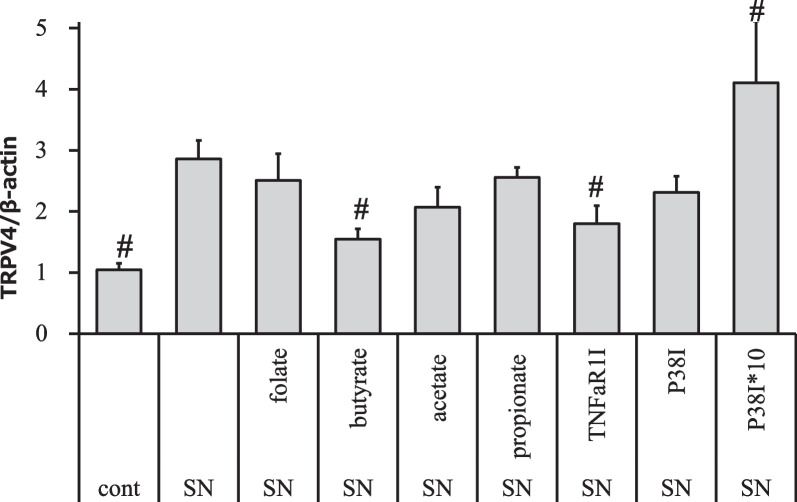
Effect of TNF-alpha inhibitors on TRPV4 expression in CCD841 cells co-cultured with bacterial supernatants. Combination exposure of CCD841 cells to *E. coli* (O111) supernatant (SN) for 3 days. Butyrate or TNFaR1 inhibitor (TNFaR1I) inhibited TRPV4 expression induced by SN exposure, but the p38 inhibitor SB202190 at 10 μM (P38I) or 100 μM (P38*10) did not. # indicate significant differences from untreated control. Error bars indicate the mean ± S.E. of five to six trials

To further investigate the regulatory mechanism of TRPV4 expression, we examined how folic acid, SFCAs, TNFαR1 inhibitor, and a p38 inhibitor affected the increase in TRPV4 expression following exposure of CCD841 cells to *E. coli* culture supernatants for 3 days. The increase was inhibited by treatment with butyrate or TNFαR1 inhibitor, but was further enhanced by treatment with p38 inhibitor (Fig. 5).

### TRPV4 overexpression in constipated patients

**Fig. 6:**
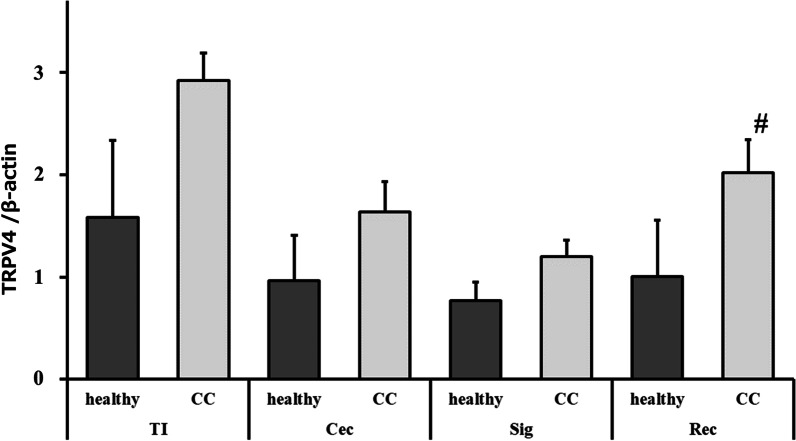
TRPV4 expression in intestinal tissues from patients with chronic constipation is increased relative to healthy controls. Patients with chronic constipation (CC, n = 5) have increased TRPV4 expression in the terminal ileum (TI), cecum (Cec), sigmoid colon (Sig), and rectum (Rec) compared to healthy subjects (n = 9); the increase in the rectum was significant (#*p* < 0.05; n = 40). Error bars indicate the mean ± S.E

Comparison of TRPV4 expression in the terminal ileum, cecum, sigmoid colon, and rectum of patients with constipation (n = 5) and healthy subjects (n = 9) revealed that TRPV4 expression was significantly increased in the rectum of patients with constipation, although differences were also observed in the terminal ileum (Fig. 6). As such, additional specimens were collected only from the rectum and analyzed for 40 subjects. Table 1 shows TRPV4 expression, male rate, age, Bristol scale (BS), constipation severity score (CSS) (total and sub scores), and ratio of rectum-adhering bacteria (%) for healthy subjects and constipated patients using ROME IV criteria. Since most women have experienced menopause by the late 50 s, data for women aged 56–86 years-old (mean 69.9 years-old) were used to minimize hormonal effects. The study included 68% men and the remaining 32% were assumed to be postmenopausal women. In the healthy group, 67.8% were male with a mean age of 68.7 years-old, a mean total CSS of 1.64, and a mean BS of 3.9. In the constipation group, 77.8% were male, with a mean age of 75.4 years-old, a mean total CSS of 9.56, and a mean BS of 3.1 (overall total CSS mean 3.6 and BS mean 3.5). Spearman correlation coefficients (Table 2) showed that BS and total CSS were inversely correlated, and TRPV4 expression was correlated with CSS sub8 (duration of constipation). Several factors were found to be correlated with the CSS subscore (Table 3). B and p values indicating statistical significance (*p* < 0.05) for each CSS subscore in a multiple regression analysis indicated that CSS sub1 (rare stool frequency), CSS sub6 (assistance for evacuation), and CSS sub8 (duration of constipation) were associated with TRPV4 expression; CSS sub3 (incomplete evacuation), CSS sub5 (length of time per attempt) and CSS7 (unsuccessful evacuation attempts per 24 h) were correlated with *E. faecalis*. No difference in TRPV4 expression and constipation symptoms due to *E. coli* O antigen was observed (data not shown).

**Table 1:** Background information for study participants (n = 40)

	Healthy		Chronic constipation	
	Average	S.E	Average	S.E
TRPV4 expression	1.00	0.13	2.02	0.45
Gender (M:1 F:0)	0.68	0.08	0.78	0.14
Age (yr)	68.71	1.76	75.44	3.32
BS	3.90	0.12	3.11	0.53
Total CSS	1.65	0.43	9.56	1.06
CSS sub1	0.03	0.03	1.22	0.44
CSS sub2	0.42	0.16	2.67	0.38
CSS sub3	0.58	0.17	0.89	0.43
CSS sub4	0.10	0.07	0.44	0.28
CSS sub5	0.26	0.14	0.56	0.28
CSS sub6	0.06	0.04	0.89	0.10
CSS sub7	0.00	0.00	0.44	0.17
CSS sub8	0.19	0.13	2.44	0.42
*P. acnes*	2.64	0.97	2.77	2.38
*E. coli*	9047.51	5696.22	13,249.54	11,671.13
*K. oxytoca*	140.95	133.47	0.05	0.04
*E. faecalis*	2403.95	2226.10	0.02	0.02
*C. perfringens*	40.98	38.22	2207.13	1946.51
*S. aureus*	16.16	6.21	12.48	10.98

**Table 2:** Correlation coefficient between each factor

	Gender	Age	BS	CSS	CSS	CSS	CSS	CSS	CSS	CSS	CSS	CSS	TRPV4	*P. acnes*	*E. coli*	*K. oxitoca*	*C. perfringens*	*E. feacalis*	*S. aureus*
				sub1	sub2	sub3	sub4	sub5	sub6	sub7	sub8	total							
Gender	1																		
Age	0.024	1																	
BS	− 0.011	0.078	1																
CSS																			
sub1	− 0.008	0.413^**^	− 0.103	1															
sub2	− 0.008	0.19	− 0.527^**^	0.272	1														
sub3	0.014	− 0.097	− 0.314^*^	− 0.119	0.155	1													
sub4	− 0.136	− 0.1	− 0.366^*^	0.301	0.124	0.407^**^	1												
sub5	0.3	0.202	− 0.273	0.162	0.340^*^	0.275	0.082	1											
sub6	0	0.19	− 0.400^*^	0.576^**^	0.433^**^	− 0.096	0.202	0.189	1										
sub7	0.036	0.004	− 0.427^**^	0.326^*^	0.486^**^	0.161	0.173	0.273	0.385^*^	1									
sub8	0.067	0.255	− 0.403^**^	0.561^**^	0.633^**^	0.047	0.339^*^	0.349^*^	0.761^**^	0.473^**^	1								
total CSS	0.005	0.221	− 0.468^**^	0.463^**^	0.745^**^	0.466^**^	0.402^*^	0.490^**^	0.585^**^	0.461^**^	0.787^**^	1							
TRPV4	0.142	0.101	0.136	0.294	0.02	− 0.252	0.031	0.055	0.28	0.188	0.381^*^	0.038	1						
*P. acnes*	0.360^*^	− 0.256	0.202	− 0.009	− 0.201	− 0.021	− 0.059	0.129	0.029	0.122	− 0.112	− 0.116	0.009	1					
*E. coli*	0.548^**^	− 0.245	0.371^*^	− 0.186	− 0.295	− 0.054	− 0.265	− 0.084	− 0.271	− 0.033	− 0.316	− .371^*^	− 0.14	0.607^**^	1				
*K. oxitoca*	0.219	0.1	− 0.082	− 0.078	− 0.05	− 0.219	0.01	− 0.015	0.103	− 0.057	0.011	− 0.237	0.163	0.308	0.162	1			
*C. perfringens*	0.217	− 0.051	0.067	0.179	− 0.135	− 0.243	− 0.026	0.073	0.252	0.107	0.07	− 0.125	0.255	0.558^**^	0.22	0.542^**^	1		
*E. faecalis*	0.311	− 0.115	− 0.04	− 0.139	− 0.1	− 0.04	− 0.048	0.328^*^	0.159	− 0.024	− 0.069	− 0.046	− 0.162	0.462^**^	0.203	0.298	0.411^*^	1	
*S. aureus*	0.183	− 0.035	0.289	− 0.097	− 0.267	0.105	− 0.218	0.068	− 0.26	− 0.024	− 0.379^*^	− 0.148	− 0.389^*^	0.616^**^	0.607^**^	− 0.099	− 0.041	0.09	1

^**^Correlation coefficients are significant (two-sided) at the 1% level

^*^Correlation coefficient is significant (two-sided) at the 5% level

**Table 3:** Results of multiple regression analysis of CSS sub-score, TRPV4 expression, and intestinal bacterial ratio

		Unstandardized coefficient	Significant probability	Confidence interval	
		B		Lower limit	Upper limit
Sub1	TRPV4 expression	0.450	0.00	0.242	0.657
	age	0.222	0.033	0.002	0.042
Sub3	*E. faecalis*	0.000	0.028	0.000	0.000
Sub5	*E. faecalis*	0.000	0.031	0.000	0.000
Sub6	TRPV4 expression	0.173	0.009	0.045	0.302
	*C. perfringens*	1.362E−5	0.028	0.000	0.000
Sub7	*E. faecalis*	5.146E−5	0.002	0.000	0.000
Sub8	TRPV4 expression	0.574	0.004	0.193	0.956

## Discussion

Unlike *H. pylori*, which induced TRPV4 methylation in the gastric epithelium [18, 45], here the intestinal bacteria and metabolites examined did not induce TRPV4 methylation in colonic epithelium. We thus tested for changes in TRPV4 expression using whole stool material from constipated patients and healthy subjects, but since quantification of concentrations in stool supernatants (N = 6) was not established, comparisons between samples could not be made. Upon observing that TRPV4 in the colon epithelium of constipated patients was increased, we examined the intestinal bacteria that induce increased TRPV4 expression as well as the mechanism associated with this increase and its relationship to clinical symptoms. Both clinical and standard strains of *E. faecalis*, *K. oxytoca*, and *E. coli* (O127, O111) increased TRPV4 expression (Fig. 1), and for *K. oxytoca* and *E. coli* the culture supernatant, but not the bacterial components, was associated with increased TRPV4 expression. In contrast, for *E. faecalis*, both bacterial components and bacterial supernatant slightly increased TRPV4 expression. In this study, TNFα alone did not enhance TRPV4 expression, whereas in *E. coli* supernatants, microarray network analysis and quantitative PCR results showed that TNFα signaling and TRPV4 expression were enhanced (Figs. 2, 3, Additional file 1: Fig. S2). TNFα signaling induces TRPV4 expression in other cell types such as dental pulp cells in which changes in TNFα-dependent effects on TRPV4 expression are manifested via the TNFR1 and p38 MAPK signaling pathways [12]. In articular cartilage, the changes are mediated via the Erk and p38 MAPK signaling pathways [13, 14]. In the present study, Erk or JNK pathways other than the p38 pathway may affect TRPV4 levels, as the changes were inhibited by TNFαR1 inhibitors, but not by p38 inhibitors (Fig. 5). TNFα expression is known to be inhibited by butyrate [15, 16], and the results of the present study suggest that the ability of butyrate to inhibit enhanced TRPV4 expression associated with *E. coli* culture supernatant co-culture may involve inhibition of TNFα expression.

TRPV4 expression was increased by an as yet unknown component in the culture supernatant, suggesting that butyrate may suppress the increase in TRPV4 expression induced by these other components, although butyrate did not directly affect the increase or decrease in TRPV4. Next, we examined the relationship between constipation symptoms and TRPV4 expression and bacteria adhering to the rectal mucosa using clinical information and human specimens from constipation patients and healthy subjects. *E. faecalis* frequency and TRPV4 expression were associated with some CSS subscores. *E. faecalis* belonging to Firmicutes (Lactobacillus), which shows a decline in frequency in the elderly, does not produce butyrate, and its relative increase in Lactobacillus may increase TRPV4 expression and cause intestinal dyssensitivity, although this potential causal relationship requires further investigation.

The results of this study appear to support a relationship between constipation symptoms and increased, rather than suppressed, TRPV4 expression. Based on the delayed gastric emptying seen for whole body TRPV4-deficient mice [45], and an increase in endogenous TRPV4 ligand in human colon tissue that correlates with diarrhea symptoms in IBS-D [46], the increased TRPV4 expression in the colon epithelium could promote colon migration. Thus, increased TRPV4 expression may be a compensatory mechanism for delayed colonic migration caused by other conditions. On the other hand, increased TRPV4 expression in the colon epithelium may be part of the pathophysiology of constipation, as the colon epithelium is chronically receptive to advancement stimuli and thus cannot accept additional pressure stimulation from the stool mass, which results in low sensitivity. Future studies should prospectively examine changes in TRPV4 expression in relation to constipation symptoms in the same patients.

This exploratory study has some limitations including a small number of subjects, the small number of bacterial species examined, the lack of intestinal susceptibility testing, and the lack of menopausal information. Many females in the study had reached menopausal age, and most were thus presumed to be menopausal. Future studies should consider an expanded panel of bacterial species along with intestinal susceptibility testing in a larger cohort of patients.

## Conclusions

Treatment with supernatants of cultures of some intestinal bacteria can increase TRPV4 expression in the colon epithelium. Increased TRPV4 expression and frequency of *E. faecalis* are associated with constipation symptoms, suggesting that butyrate or TNFαR1 inhibition may inhibit increased TRPV4 expression.

## Supplementary Information

**Additional file 1. Figure S1.** Pyrosequencing of the TRPV4 gene in CCD841 cells exposed to *P. acnes*, *S. aureus* or *C. perfringens* for 1 day. Each point represents the percent methylation of the TRPV4 gene in CCD841 in co-culture with each bacterium, and the lines represent the mean and range. No abnormalities in methylation were detected. **Figure S2.** A. Microarray network analysis of CCD841 cells using the KEGG pathway database. Human gene names are shown in green text. Genes having increased and decreased expression in the array are colored red and blue, respectively. Treatment of cells with *E. coli* (O111) SN showed activation of TNFR2 signaling, cytokine and NOD signals. TNF signaling pathway induces TNFR2 expression and leukocyte recruitment. Expression of TNF family members is induced in cytokine receptor association. Pro-inflammatory cytokines were also induced in the NOD-like receptor signaling pathway. Kanehisa laboratory kindly allowed us to cite the KEGG pathway map.

**Additional file 2. Table S1.** Clinical isolates and Standard isolates of Enterobacteriaceae used in this study. **Table S2.** Primer sequences for qPCR.

**Additional file 3.** The original untreated full-length blots of TRPV4, TNFαR2 and βactin are presented with molecular size markers. GES-1 was used as a positive control to confirm that TRPV4 was specifically detected.

## Data Availability

The datasets are available in the GEO repository, https://www.ncbi.nlm.nih.gov/geo/query/acc.cgi?acc=GSE208164.

## Abbreviations

TRPV4: Transient receptor potential vanilloid 4
LPS: Lipopolysaccharide
SCFA: Short-chain fatty acid
