# Supplementary material for: Colonic TRPV4 overexpression is related to constipation severity
Source: BMC Gastroenterol. 2023 Jan 13;23:13. doi: 10.1186/s12876-023-02647-0 (PMC9838009; doi:10.1186/s12876-023-02647-0)
Supplement: Supplementary file 1 — Additional file 1. Figure S1. Pyrosequencing of the TRPV4 gene in CCD841 cells exposed to P. acnes, S. aureus or C. perfringens for 1 day. Each point represents the percent methylation of the TRPV4 gene in CCD841 in co-culture with each bacterium, and the lines represent the mean and range. No abnormalities in methylation were detected. Figure S2. A. Microarray network analysis of CCD841 cells using the KEGG pathway database. Human gene names are shown in green text. Genes having increased and decreased expression in the array are colored red and blue, respectively. Treatment of cells with E. coli (O111) SN showed activation of TNFR2 signaling, cytokine and NOD signals. TNF signaling pathway induces TNFR2 expression and leukocyte recruitment. Expression of TNF family members is induced in cytokine receptor association. Pro-inflammatory cytokines were also induced in the NOD-like receptor signaling pathway. Kanehisa laboratory kindly allowed us to cite the KEGG pathway map. [file 12876_2023_2647_MOESM1_ESM.pptx]

## Slide 1
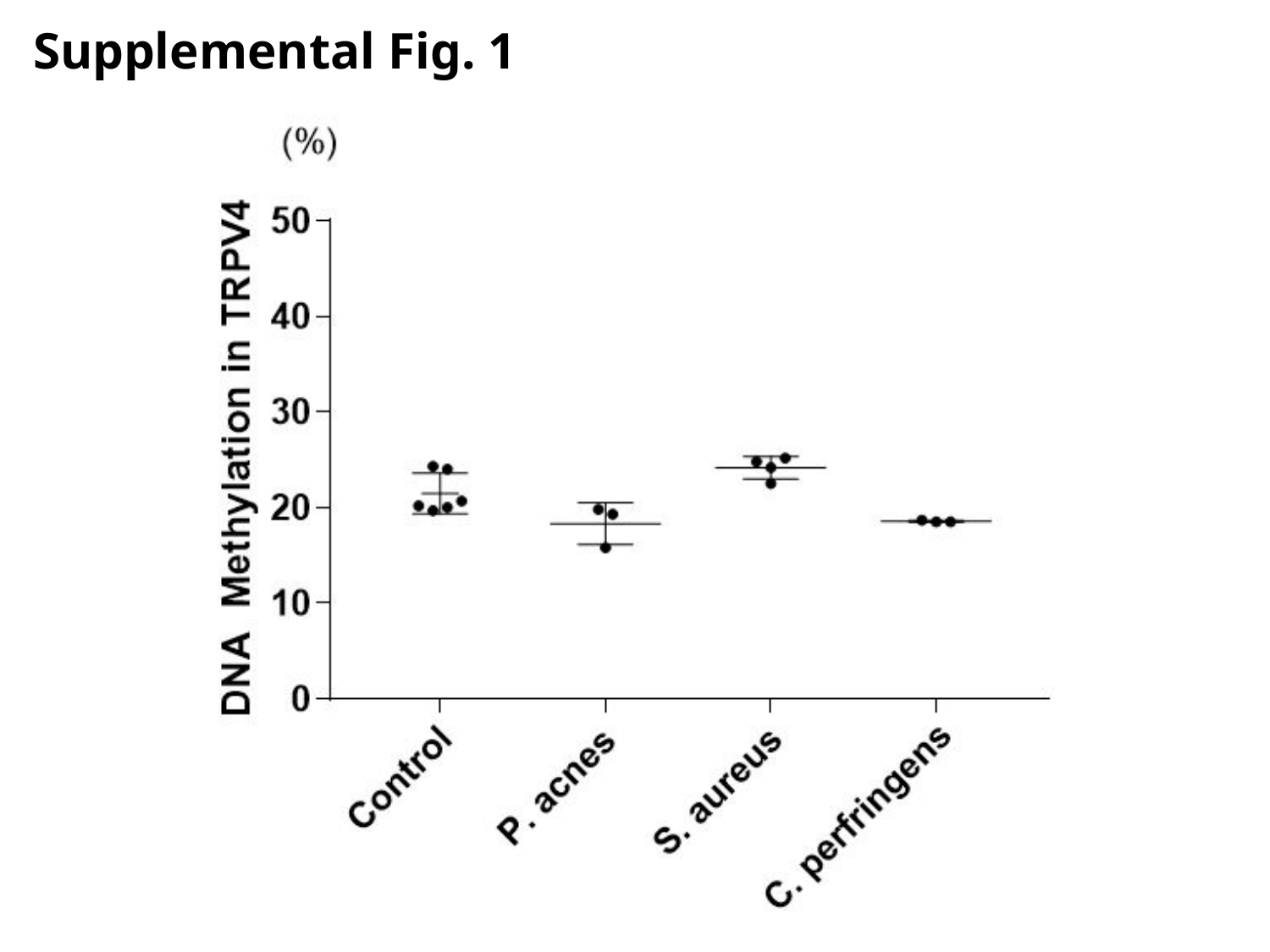

Supplemental Fig. 1

## Slide 2
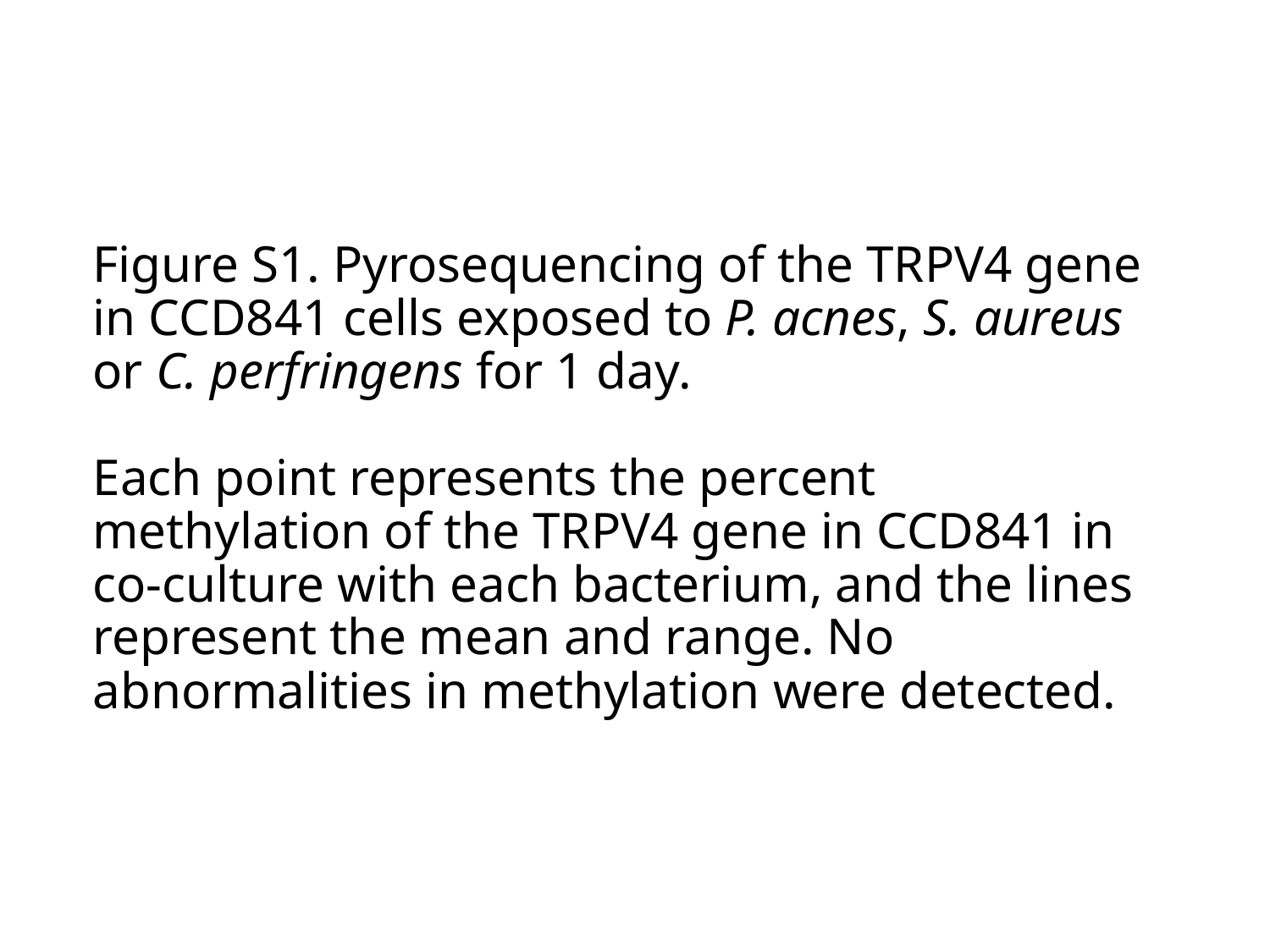

# Figure S1. Pyrosequencing of the TRPV4 gene in CCD841 cells exposed to P. acnes, S. aureus or C. perfringens for 1 day. Each point represents the percent methylation of the TRPV4 gene in CCD841 in co-culture with each bacterium, and the lines represent the mean and range. No abnormalities in methylation were detected.

## Slide 3
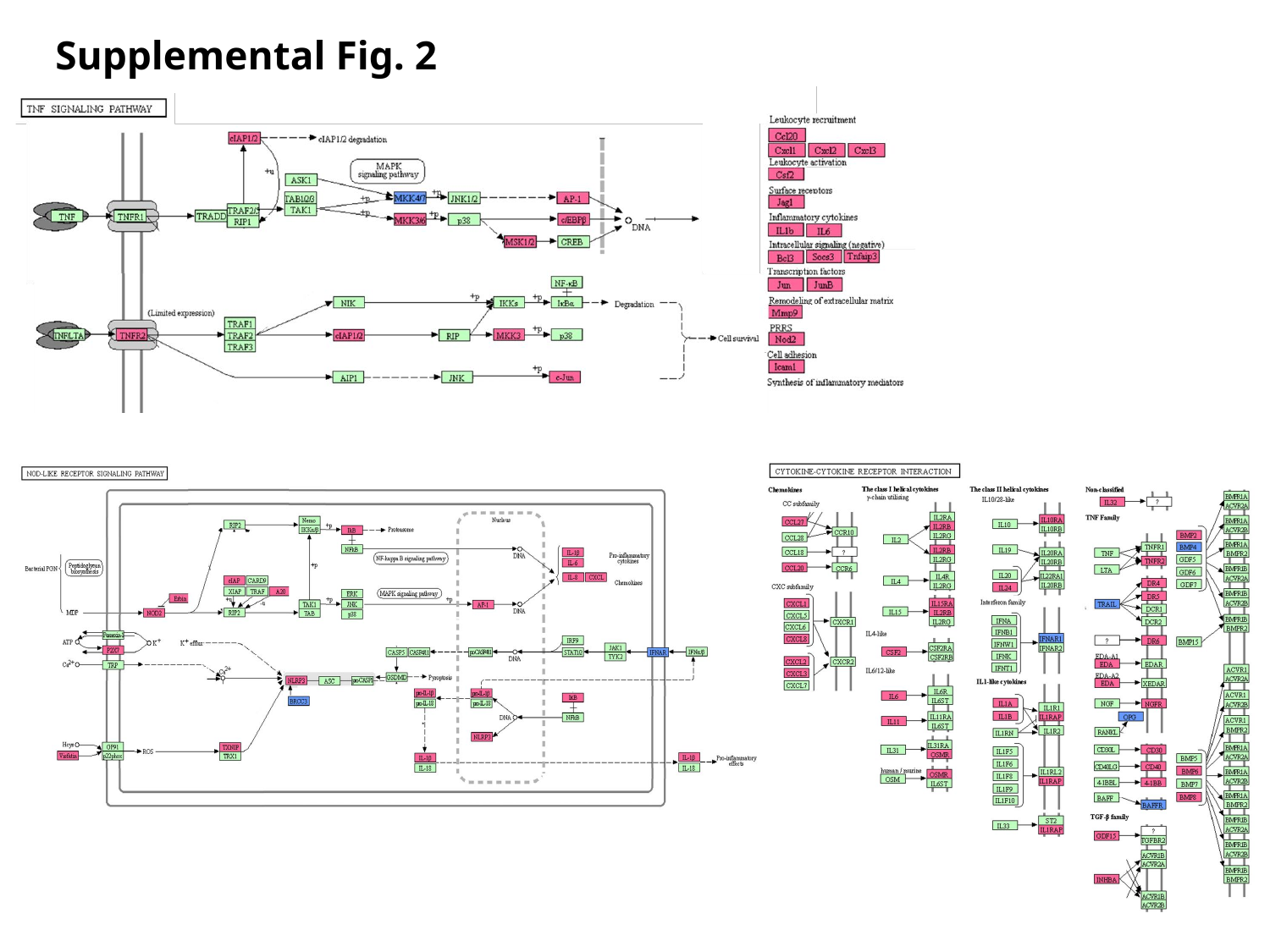

Supplemental Fig. 2

## Slide 4
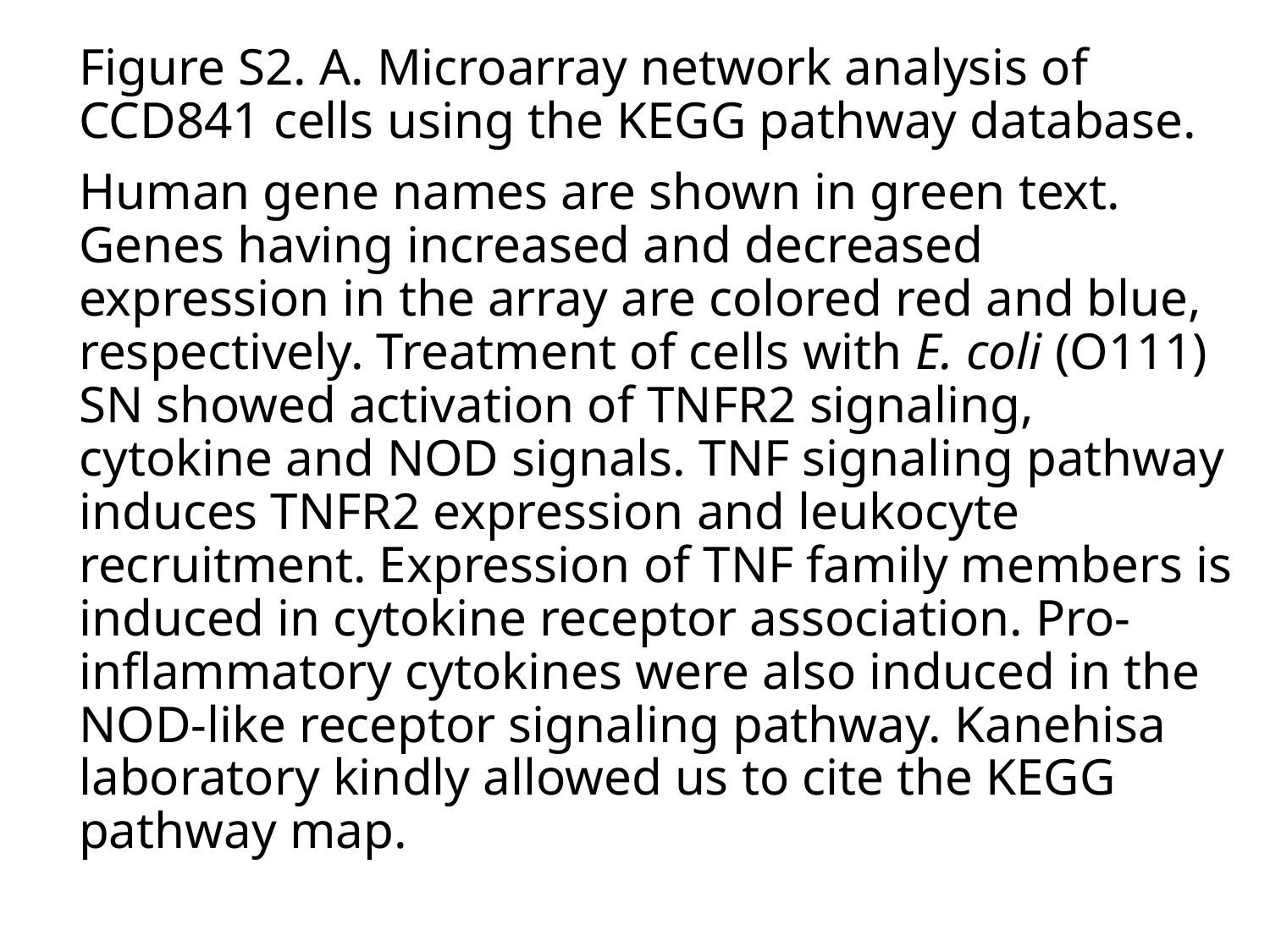

Figure S2. A. Microarray network analysis of CCD841 cells using the KEGG pathway database.
Human gene names are shown in green text. Genes having increased and decreased expression in the array are colored red and blue, respectively. Treatment of cells with E. coli (O111) SN showed activation of TNFR2 signaling, cytokine and NOD signals. TNF signaling pathway induces TNFR2 expression and leukocyte recruitment. Expression of TNF family members is induced in cytokine receptor association. Pro-inflammatory cytokines were also induced in the NOD-like receptor signaling pathway. Kanehisa laboratory kindly allowed us to cite the KEGG pathway map.
