# Supplementary material for: Colonic TRPV4 overexpression is related to constipation severity
Source: BMC Gastroenterol. 2023 Jan 13;23:13. doi: 10.1186/s12876-023-02647-0 (PMC9838009; doi:10.1186/s12876-023-02647-0)
Supplement: Supplementary file 2 — Additional file 2. Table S1. Clinical isolates and Standard isolates of Enterobacteriaceae used in this study. Table S2. Primer sequences for qPCR. [file 12876_2023_2647_MOESM2_ESM.docx]

**Table S1.** Clinical isolates and Standard isolates of Enterobacteriaceae used in this study.

| Clinical isolates | Standard isolates |
| --- | --- |
| *Propionibacterium acnes* | JCM No 6495 *Cutibacterium acnes* subsp. *acnes* (Gilchrist 1900) Nouioui et al. 2018 |
| *Clostridium perfringens* | JCM No *Clostridium perfringens* (Veillon and Zuber 1898) Hauduroy et al. 1937 1 |
| *Staphylococcus aureus* | RIMD 3109015 *Staphylococcus aureus* |
| *Lactobacillus* | RIMD 3112001 *Staphylococcus aureus* |
| *Fusobacterium nucleatum* | RIMD 3116001 *Enterococcus faecalis* |
| *Bifidobacteria* | JCM No 1665T *Klebsiella oxytoca* (Flügge 1886) Lautrop 1956 |
| *Clostridium butyricum* | RIMD 0509486 *Escherichia coli* (O127) |
| *Bacteroides fragilis* | RIMD 05092028 *Escherichia coli* (O111) |
| *Ruminococus* | RIMD 05092027 *Escherichia coli* (O111) |
| *Klebsiella pneumoniae* | RIMD 0509418 *Escherichia coli* (O55) |
| *Streptococcus constellatus* |  |
| *Klebsiella oxytoca* |  |
| *Enterococcus faecalis* |  |
| *Escherichia coli* |  |

**Table S2.** Primer sequences for qPCR

hTRPV4-F TCATGATCGGCTACGCTTCA

hTRPV4-R CCTCATTGCACACCTTCATGTT

hTNFa-F AGCCCATGTTGTAGCAAACC

hTNFa-R TGAGGTACAGGCCCTCTGAT

hTNFR2-F GGTGACTGTCCCAACTTTGC

hTNFR2-R GGGTCATCAGTGTCTAGGCT

hTNFR1-F CTTCGCTCTTCCAGTTGG

hTNFR1-R AGGCAAGTGAGGCACCTT

hIL-6-F AGACAGCCACTCACCTCTTCAG

hIL-6-R TTCTGCCAGTGCCTCTTTGCTG

hNOD2-F GCACTGATGCTGGCAAAGAACG

hNOD2-R CTTCAGTCCTTCTGCGAGAGAAC

hMMP2-F AGCGAGTGGATGCCGCCTTTAA

hMMP2-R CATTCCAGGCATCTGCGATGAG

hMMP6-F GAGCACTGGTACTTGGATTTGCC

hMMP6-R TTCTCAACCTCAGGATTAAATCCTC

hMMP8-F CAACCTACTGGACCAAGCACAC

hMMP8-R TGTAGCTGAGGATGCCTTCTCC

hIL-1b-F CCACAGACCTTCCAGGAGAATG

hIL-1b-R GTGCAGTTCAGTGATCGTACAGG

hIL-1a-F TGTATGTGACTGCCCAAGATGAAG

hIL-1a-R AGAGGAGGTTGGTCTCACTACC

hTRAIL-F TGGCAACTCCGTCAGCTCGTTA

hTRAIL-R AGCTGCTACTCTCTGAGGACCT

hb-actin-F TGGCACCCAGCACAATGAA

hb-actin-R CTAAGTCATAGTCCGCCTAGAAGCA

**Table S3.** Primary secondary antisera for western blotting.

**Tissue antigen Host Dilution Source (Reference)**

**Anti-TNF Receptor II Rabbit 1:250 Abcam (ab109322)**

**Anti-TRPV4 Rabbit 1:500 Abcam (ab39260)**

**Table S4.** Primer sequences for TRPV4 pyrosequencing.

PCR amplicon

Primer Temp (bp) CG sites

sense GTTTYGGAGGTAAGTGGGG 60 ˚C (35) 129 6

anti-sense CCCCTAACCCRCATTTTAAAACC

sequence GGAGGTAAGTGGGGT

sequence to analyze

TYGGGTTYGGGAGGGGYGGTTTAGTYGAGGTTTTTTYGYGTTTTTGGGGATATTTT

**Table S5.** Bacteria-specific primers.

PA-F GCGTGAGTGACGGTAATGGGTA

PA-R TTCCG ACGCGATCAACCA

EC-F CATGCAAGTCGAACGGTAACAG

EC-R GCGACGTTATGCGGTATTAGC

PEH-C GATAC-GGAGTATGCCTTTACGGTG

PEH-D TAGCCTTTATCAAGCGGATACTGG

CP-F AAAGATGGCATCATCATTCAAC

CP-R TACCGTCATTATCTTCCCCAA

Efaec-F GTTTATGCCGCATGGCATAAGAG

Efaec-R CCGTACGGGGACGTTCAG

femA-SA-F TGCCTTTACAGATAGCATGCCA

femA-SA-R AGTAAGTAAGCAAGCTGCAATGACC

16SrRNA-F GAGTTTGATCMTGGCTCAG

16SrRNA-R GTATTACCGCGGCKGCTG

| **Table S6.** Genes related to *E. coli* supernatants identified in microarray analysis | | | | |
| --- | --- | --- | --- | --- |
| GeneSymbol | Description | Accession No. | Zscore | Ratio |
| **TNFalpha signaling** |  |  |  |  |
| BIRC2 | *Homo sapiens* baculoviral IAP repeat containing 2 (BIRC2) | NM_001166 | 1.718237 | 2.134781 |
| BIRC3 | *Homo sapiens* baculoviral IAP repeat containing 3 (BIRC3) | NM_001165 | 3.712737 | 7.403554 |
| MAP2K3 | *Homo sapiens* mitogen-activated protein kinase kinase 3 (MAP2K3) | NM_001316332 | 2.409918 | 2.432628 |
| MAP2K4 | *Homo sapiens* mitogen-activated protein kinase kinase 4 (MAP2K4) | NM_003010 | -2.19677 | 0.420991 |
| MAP2K6 | *Homo sapiens* mitogen-activated protein kinase kinase 6 (MAP2K6) | NM_002758 | -1.81513 | 0.332923 |
| CCL20 | *Homo sapiens* C-C motif chemokine ligand 20 (CCL20) | NM_004591 | 3.188747 | 17.92305 |
| CXCL1 | *Homo sapiens* C-X-C motif chemokine ligand 1 (CXCL1) | NR_046035 | 4.229624 | 6.507782 |
| CXCL2 | *Homo sapiens* C-X-C motif chemokine ligand 2 (CXCL2) | NM_002089 | 2.68623 | 4.256304 |
| CXCL3 | C-X-C motif chemokine ligand 3 | ENST00000296026 | 3.414055 | 7.89961 |
| JAG1 | *Homo sapiens* jagged canonical Notch ligand 1 (JAG1) | NM_000214 | 2.248187 | 3.899289 |
| IL1B | interleukin 1 beta | ENST00000263341 | 7.861423 | 22.5255 |
| IL6 | *Homo sapiens* interleukin 6 (IL6) | NM_000600 | 5.865388 | 23.63688 |
| BCL3 | *Homo sapiens* BCL3 transcription coactivator (BCL3) | NM_005178 | 2.738997 | 2.746729 |
| SOCS3 | *Homo sapiens* suppressor of cytokine signaling 3 (SOCS3) | NM_003955 | 2.46493 | 2.973591 |
| TNFAIP3 | *Homo sapiens* TNF alpha induced protein 3 (TNFAIP3) | NM_006290 | 3.924274 | 8.298163 |
| JUN | Jun proto-oncogene, AP-1 transcription factor subunit | ENST00000371222 | 2.334762 | 2.529135 |
| JUNB | *Homo sapiens* JunB proto-oncogene, AP-1 transcription factor subunit (JUNB) | NM_002229 | 2.09144 | 3.088398 |
| MMP9 | *Homo sapiens* matrix metallopeptidase 9 (MMP9) | NM_004994 | 5.441849 | 18.81037 |
| NOD2 | *Homo sapiens* nucleotide binding oligomerization domain containing 2 (NOD2) | NM_022162 | 2.186594 | 7.364664 |
| ICAM1 | *Homo sapiens* intercellular adhesion molecule 1 (ICAM1) | NM_000201 | 4.166115 | 5.220019 |
| TNFRSF1B | *Homo sapiens* TNF receptor superfamily member 1B (TNFRSF1B) | NM_001066 | 3.022076 | 6.230407 |
| **NOD-2** |  |  |  |  |
| ERBIN | *Homo sapiens* erbb2 interacting protein (ERBIN) | NM_018695 | 2.274219 | 2.31381 |
| TNFAIP3(A20) | *Homo sapiens* TNF alpha induced protein 3 (TNFAIP3) | NM_006290 | 3.924274 | 8.298163 |
| CXCL8 | *Homo sapiens* C-X-C motif chemokine ligand 8 (CXCL8) | NM_000584 | 8.214995 | 144.6239 |
| P2RX7 | *Homo sapiens* purinergic receptor P2X 7 (P2RX7) | NM_002562 | 2.5171 | 3.043248 |
| P2RX7 | *Homo sapiens* purinergic receptor P2X 7 (P2RX7) | NM_002562 | 2.655305 | 3.235763 |
| NLRP3 | *Homo sapiens* NLR family pyrin domain containing 3 (NLRP3) | NM_001079821 | 3.988236 | 11.18443 |
| IFNAR1 | *Homo sapiens* interferon alpha and beta receptor subunit 1 (IFNAR1) | NM_000629 | -2.69996 | 0.233126 |
| IFNAR2 | *Homo sapiens* interferon alpha and beta receptor subunit 2 (IFNAR2) | NM_000874 | 1.63451 | 1.917081 |
| TXNIP | *Homo sapiens* thioredoxin interacting protein (TXNIP) | NM_001313972 | 6.4619 | 5.526457 |
| **Cytokines** |  |  |  |  |
| CCL20 | *Homo sapiens* C-C motif chemokine ligand 20 (CCL20) | NM_004591 | 3.188747 | 17.92305 |
| CCL27 | C-C motif chemokine ligand 27 | ENST00000557161 | 2.998528 | 15.13896 |
| CSF2 | *Homo sapiens* colony stimulating factor 2 (CSF2) | NM_000758 | 5.803024 | 33.5659 |
| IL2RB | *Homo sapiens* interleukin 2 receptor subunit beta (IL2RB) | NM_000878 | 3.155021 | 5.48054 |
| IL15RA | *Homo sapiens* interleukin 15 receptor subunit alpha (IL15RA) | NM_172200 | 2.098035 | 3.099401 |
| IL11 | *Homo sapiens* interleukin 11 (IL11) | NM_000641 | 3.077417 | 3.393048 |
| OSMR | *Homo sapiens* oncostatin M receptor (OSMR) | NM_001323504 | 3.7556 | 4.437404 |
| IL10RA | *Homo sapiens* interleukin 10 receptor subunit alpha (IL10RA) | NM_001558 | 2.430887 | 9.147674 |
| IL24 | *Homo sapiens* interleukin 24 (IL24) | NM_001185156 | 6.24949 | 43.98634 |
| IL32 | *Homo sapiens* interleukin 32 (IL32) | NM_001012631 | 4.423129 | 7.091398 |
| IL1RAP | *Homo sapiens* interleukin 1 receptor accessory protein (IL1RAP) | NM_134470 | 2.580797 | 3.130511 |
| IL1A | *Homo sapiens* interleukin 1 alpha (IL1A) | NM_000575 | 4.829774 | 18.61814 |
| IL1B | interleukin 1 beta | ENST00000263341 | 7.861423 | 22.5255 |
| IFNAR1 | *Homo sapiens* interferon alpha and beta receptor subunit 1 (IFNAR1) | NM_000629 | -2.69996 | 0.233126 |
| EDA | *Homo sapiens* ectodysplasin A (EDA) | NM_001005610 | 2.032968 | 3.422815 |
| NGFR | *Homo sapiens* nerve growth factor receptor (NGFR) | NM_002507 | 5.629302 | 20.81128 |
| CD40 | *Homo sapiens* CD40 molecule (CD40) | NM_001302753 | 4.010125 | 11.33367 |
| INHBA | *Homo sapiens* inhibin subunit beta A (INHBA) | NM_002192 | 2.886848 | 3.585978 |
| GDF15 | *Homo sapiens* growth differentiation factor 15 (GDF15) | NM_004864 | 4.486302 | 5.234179 |
| BMP2 | *Homo sapiens* bone morphogenetic protein 2 (BMP2) | NM_001200 | 2.24976 | 3.903006 |
| BMP6 | *Homo sapiens* bone morphogenetic protein 6 (BMP6) | NM_001718 | 2.414575 | 3.676303 |
| BMP8A | *Homo sapiens* bone morphogenetic protein 8a (BMP8A) | NM_181809 | 2.926109 | 14.19657 |
| BMP8B | *Homo sapiens* bone morphogenetic protein 8b (BMP8B) | NM_001720 | 1.681611 | 2.766799 |
