# Supplementary material for: Colonic TRPV4 overexpression is related to constipation severity
Source: BMC Gastroenterol. 2023 Jan 13;23:13. doi: 10.1186/s12876-023-02647-0 (PMC9838009; doi:10.1186/s12876-023-02647-0)

TRPV4 600sec

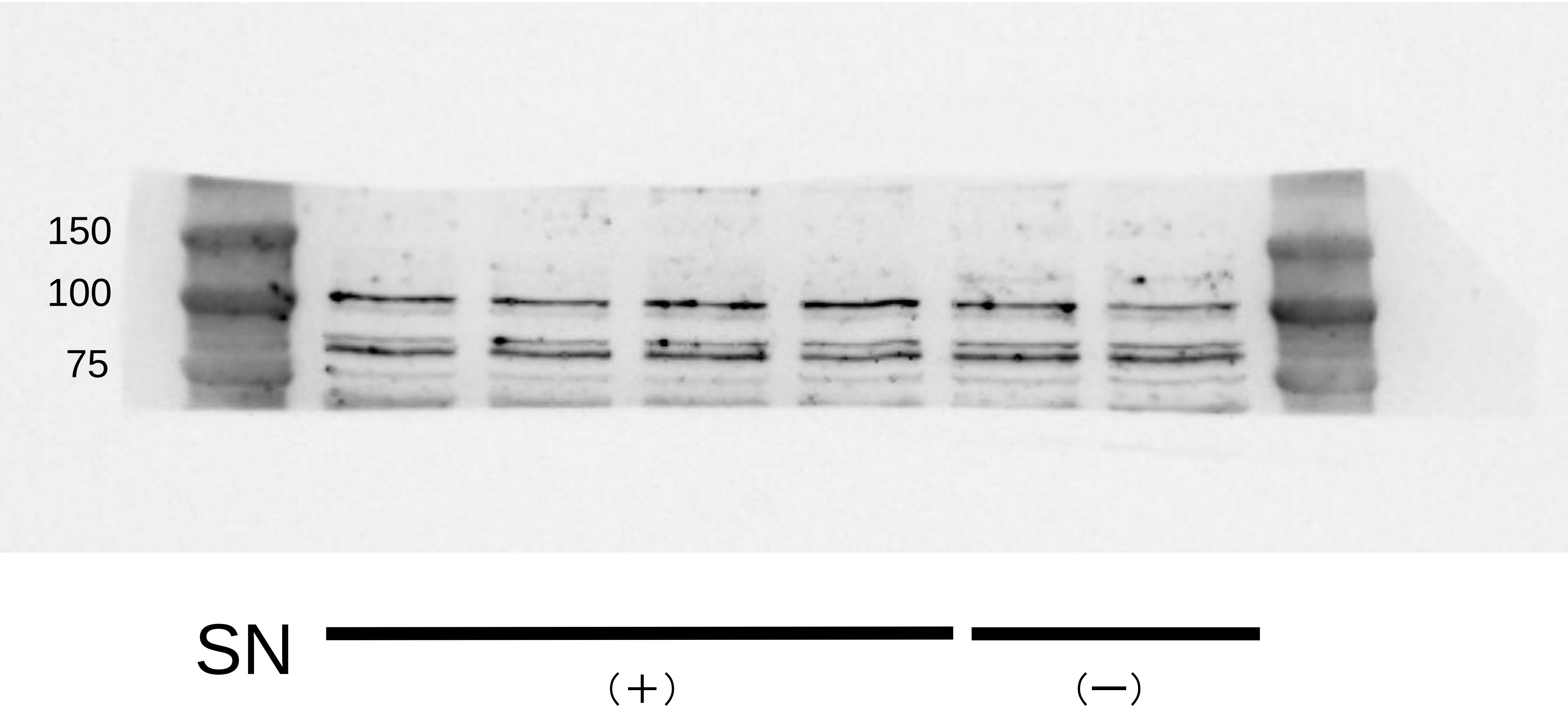

TNFαR2 increhigh20sec_1sec

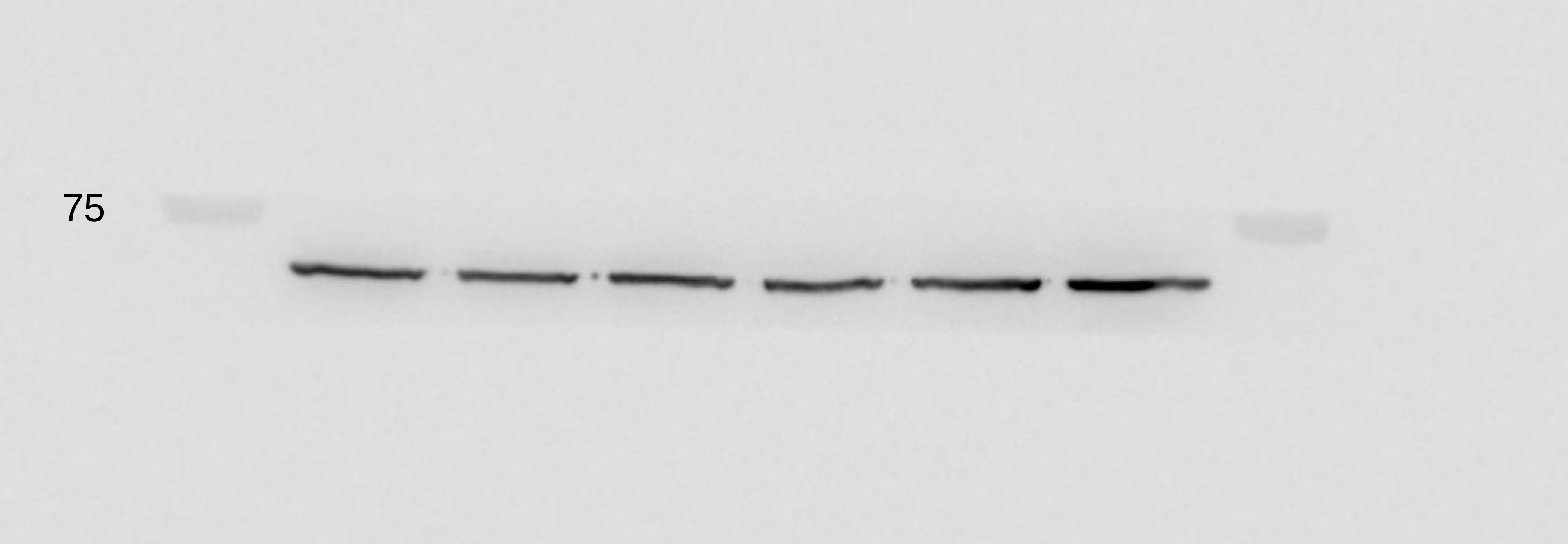

SN

(+) (−)

actinhigh120sec

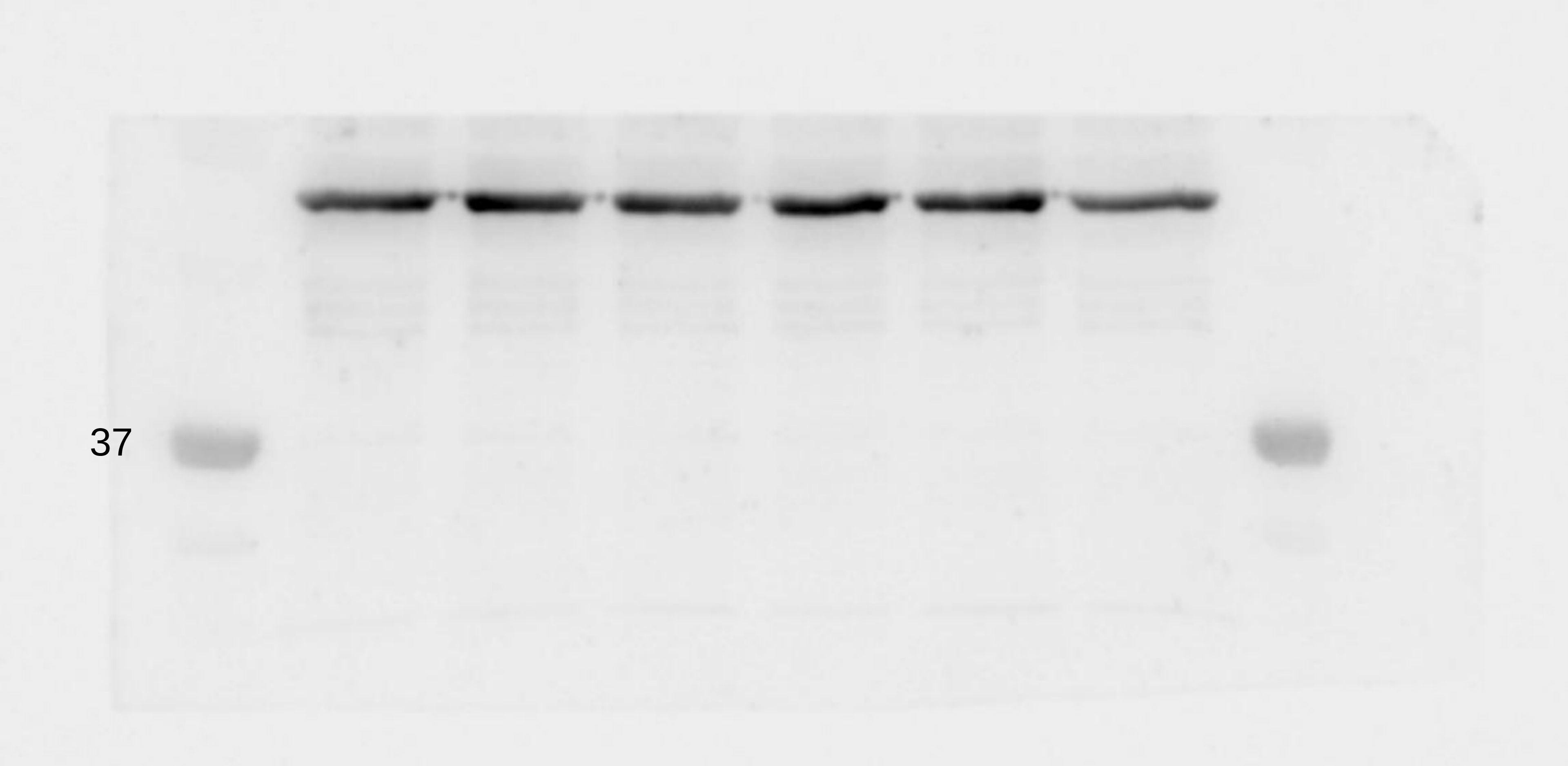

SN

（+） （−）

TRPV4 expression in gastric GES-1 cell line

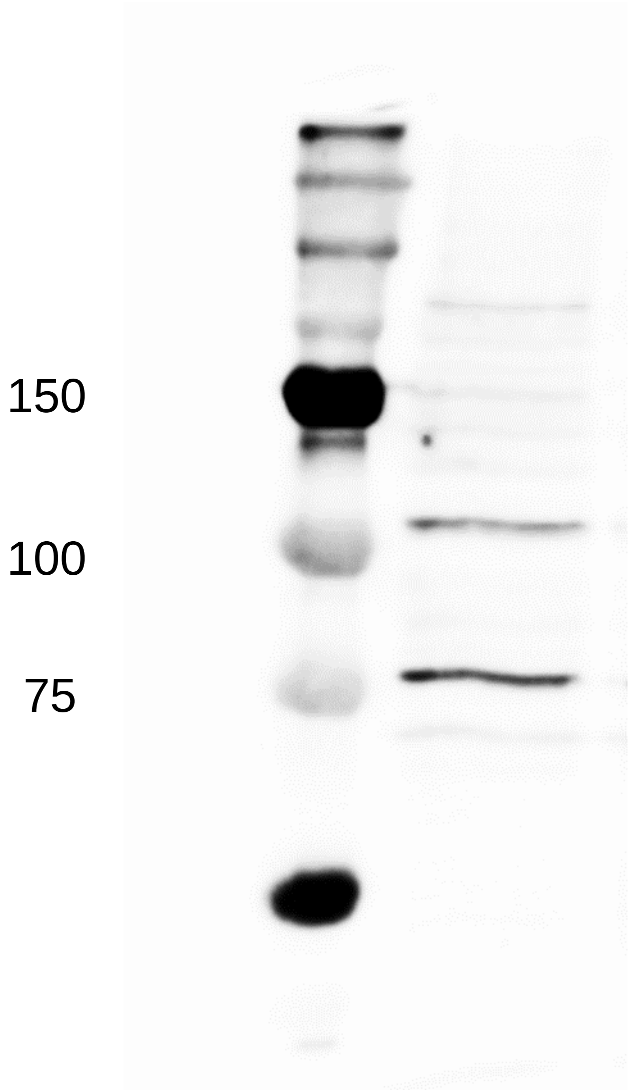

Supplement: Supplementary file 3 — Additional file 3. The original untreated full-length blots of TRPV4, TNFαR2 and βactin are presented with molecular size markers. GES-1 was used as a positive control to confirm that TRPV4 was specifically detected. [file 12876_2023_2647_MOESM3_ESM.pdf]
